# *ϕ*-Divergence in Contingency Table Analysis

**DOI:** 10.3390/e20050324

**Published:** 2018-04-27

**Authors:** Maria Kateri

**Affiliations:** Institute of Statistics, RWTH Aachen University, 52062 Aachen, Germany; maria.kateri@rwth-aachen.de

**Keywords:** log-linear models, ordinal classification variables, association models, correlation models

## Abstract

The ϕ-divergence association models for two-way contingency tables is a family of models that includes the association and correlation models as special cases. We present this family of models, discussing its features and demonstrating the role of ϕ-divergence in building this family. The most parsimonious member of this family, the model of ϕ-scaled uniform local association, is considered in detail. It is implemented and representative examples are commented on.

## 1. Introduction

Contingency tables and their analysis are of special importance for various diverse fields, like medical sciences, psychology, education, demography and social sciences. In these fields, categorical variables and their cross-classification play a predominant role, since characteristics of interest are often categorical, nominal or most frequently ordinal. For example, diagnostic ratings, strength of opinions or preferences, educational attainments and socioeconomic characteristics are expressed in ordinal scales. The origins of contingency table analysis (CTA) lie back in 1900 with the well-known contributions by Pearson and Yule, while, for the history of CTA before 1900, we refer to the interesting paper by Stigler [1]. The interest lies mainly in identifying and describing structures of underlying association in terms of appropriate models or measures. Divergence measures have been employed in the CTA mainly for hypothesis testing (model fit) and estimation, leading to general families of test statistics and estimators. The family of ϕ-divergence test statistics contain the classical likelihood ratio and Pearson test statistics as special cases while the maximum likelihood estimators (MLEs) belong to the family of the minimum ϕ-divergence estimators (MϕEs). Families of ϕ-divergence based test statistics as well as MϕEs for various standard models in CTA have a long history. However, their consideration and discussion is out of our scope. For a detailed overview and related references, we refer to the comprehensive book of Pardo [2]. For log-linear models, see also [3].

Here, we aim at highlighting a different structural role of ϕ-divergence in contingency tables modelling, namely that of linking phenomenological different models, forming thus a family of models and providing a basis for their comparison, understanding and unified treatment. Through this approach, new insight is gained for the standard association and correlation models (see [4,5]) while further alternatives are considered. We restrict our discussion on two-dimensional contingency tables, but the models and approaches discussed are directly extendable to tables of higher dimension.

The organization of the paper is as follows. Preliminaries on log-linear models, divergence measures, association and correlation models for two-way tables are provided in Section 2. In the sequel, the general family of ϕ-divergence based association models (AMs), which includes the classical association and correlation models as special cases, is reviewed in Section 3. The most parsimonious ϕ-divergence based association model, that of ϕ-scaled uniform local association, and its role in conditional testing of independence is considered and discussed in Section 4. For this family of models, the effect of the specific ϕ-function used is illustrated by analysing representative examples in Section 5. Some final comments are provided in Section 6.

## 2. Preliminaries

Consider an I×J contingency table $n=(n_{ij})$ with rows and columns classification variables *X* and *Y*, respectively, where $n_{ij}$ is the observed frequency in cell (i,j). The total sample size $n=\sum_{i,j}n_{ij}$ is fixed and the random table N is multinomial distributed N∼M(n,π), with probability table $\pi \in \Delta_{IJ}$, where $\Delta_{IJ}$ is the simplex $\Delta_{IJ}=\left\{\pi =\left(\pi_{ij}\right):\pi_{ij}>0,\;\sum_{i,j}\pi_{ij}=1\right\}$. Let m=E(N)=nπ be the table of expected cell frequencies. Since the mapping (n,π)↦m is one-to-one on $\Delta_{IJ}$, $m\in \{m=\left(m_{ij}\right):m_{ij}>0,\;\sum_{i,j}m_{ij}=n\}$ and models (hypotheses) for π can equivalently be expressed in terms of m. Furthermore, let $\pi_{r}={\left(\pi_{1+},\ldots ,\pi_{I+}\right)}^{T}$ and $\pi_{c}={\left(\pi_{+1},\ldots ,\pi_{+J}\right)}^{T}$ be the row and column marginal probabilities vectors, respectively, and $p=(p_{ij})$ the table of sample proportions with $p_{ij}=n_{ij}/n$.

The classical independence hypothesis for the classification variables *X* and *Y* ($\pi =\pi_{r}\pi_{c}^{T}=\pi^{I}$) corresponds to the log-linear model of independence (I), defined in terms of expected cell frequencies as

(1) $$\log\left(m_{ij}\right)=\lambda +\lambda_{i}^{X}+\lambda_{j}^{Y}\;,\;\;\;\;i=1,\ldots ,I,\;j=1,\ldots ,J,$$

where $\lambda_{i}^{X}$ and $\lambda_{j}^{Y}$ are the *i*-th row and *j*-th column main effects, respectively, while λ is the intercept. If the independence model is rejected, the interaction between *X* and *Y* is significant and the only alternative in the standard log-linear models set-up is the saturated model

(2) $$\log\left(m_{ij}\right)=\lambda +\lambda_{i}^{X}+\lambda_{j}^{Y}+\lambda_{ij}^{XY}\;,\;\;\;\;i=1,\ldots ,I,\;j=1,\ldots ,J,$$

which imposes no structure on π. Identifiability constraints need to be imposed on the main effect and interaction parameters of these models, like $\lambda_{1}^{X}=\lambda_{1}^{Y}=0$ and $\lambda_{1j}^{XY}=\lambda_{i1}^{XY}=0$, for all i,j.

An important generalized measure for measuring the divergence between two probability distributions is the ϕ-divergence. Let $\pi =(\pi_{ij})$, $q=(q_{ij})$$\in \Delta_{IJ}$ be two discrete finite bivariate probability distributions. Then, the ϕ–divergence between q and π (or Csiszar’s measure of information in q about π), is given by

(3) $$I_{\phi }^{C}\left(q,\pi \right)=\sum_{i,j}\pi_{ij}\phi \left(q_{ij}/\pi_{ij}\right),\;$$

where ϕ is a real–valued strictly convex function on [0,∞) with $\phi \left(1\right)=\phi^{\prime }\left(1\right)=0$, 0ϕ(0/0)=0, $0\phi \left(y/0\right)=\lim_{x\to \infty }\phi \left(x\right)/x$ (cf. [2]). Setting ϕ(x)=xlogx, (3) is reduced to the Kullback–Leibler (KL) divergence

(4) $$I^{\mathrm{KL}}\left(q,\pi \right)=\sum_{i,j}q_{ij}\log\left(q_{ij}/\pi_{ij}\right),\;$$

while, for $\phi \left(x\right)={\left(1-x\right)}^{2}$, Pearson’s divergence is derived. If $\phi \left(x\right)=\frac{x^{\lambda +1}-x}{\lambda (\lambda +1)}$, (3) becomes the power divergence measure of Cressie and Read [6]

(5) $$I_{\lambda }^{CR}\left(q,\pi \right)=\frac{1}{\lambda (\lambda +1)}\sum_{i=1}^{K}q_{i}\left[{\left(\frac{q_{i}}{\pi_{i}}\right)}^{\lambda }-1\right]\;,\;-\infty <\lambda <\infty \;,\;\;\lambda \neq -1,\;0.\;\;$$

For λ→−1 and λ→−0, (5) converges to the $I^{\mathrm{KL}}\left(\pi ,q\right)$ and $I^{\mathrm{KL}}\left(q,\pi \right)$, respectively, while λ=1 corresponds to Pearson’s divergence.  

The goodness of fit (GOF) of model (1) is usually tested by the likelihood ratio test statistic $G^{2}=2nI^{\mathrm{KL}}\left(p,\hat{\pi }\right)$ or Pearson’s $X^{2}=2nI_{1}^{CR}\left(p,\hat{\pi }\right)$, where $\hat{\pi }$ is the MLE of π under (1). Both test statistics are under (1) asymptotically $X_{\left(I-1)(J-1\right)}^{2}$ distributed. Alternatively, ϕ-divergence test statistics can be used (see [3]).

### 2.1. Association Models

In case of ordinal classification variables, the association models (AMs) impose a special structure on the underlying association and thus provide non-saturated models of dependence. AMs are based on scores $\mu =(\mu_{1},\ldots ,\mu_{I})$ and $\nu =(\nu_{1},\ldots ,\nu_{J})$ assigned to the rows and columns of the ordinal classification variables, respectively. They are defined by the expression

(6) $$\log\left(m_{ij}\right)=\lambda +\lambda_{i}^{X}+\lambda_{j}^{Y}+\zeta \mu_{i}\nu_{j}\;,\;\;\;\;i=1,\ldots ,I,\;j=1,\ldots ,J,$$

where the row and column scores are standardized subject to weights $w_{1}={\left(w_{11},\ldots ,w_{1I}\right)}^{T}$ and $w_{2}={\left(w_{21},\ldots ,w_{2J}\right)}^{T}$, respectively, i.e., it holds

(7) $$\sum_{i}w_{1i}\mu_{i}=\sum_{j}w_{2j}\nu_{j}=0\;\mathrm{and}\;\sum_{i}w_{1i}\mu_{i}^{2}=\sum_{j}w_{2j}\nu_{j}^{2}=1.$$

Usually, the uniform ($w_{1}=1_{I}$, $w_{2}=1_{J}$, where $1_{k}$ is a k×1 vector of 1s) or the marginal weights ($w_{1}=\pi_{r}$, $w_{2}=\pi_{c}$) are used.

If μ and ν are both known and ordered, then (6) has just one parameter more than independence, parameter ζ, and is known as the *Linear-by-Linear* (LL) AM. In case the vector μ is unknown, (6) is the *Row effect* (R) AM, while the *Column effect* (C) AM is defined analogously. Finally, when the row and the column scores are all unknown parameters to be estimated, (6) is the *multiplicative Row-Column* (RC) AM. Scores that are unknown need not necessarily to be ordered. Note that models LL, R and C are log-linear while the RC is not. The degrees of freedom (df) of these AMs equal df(LL)=(I−1)(J−1)−1, df(R)=(I−1)(J−2), df(C)=(I−2)(J−1) and df(RC)=(I−2)(J−2). The special LL model for which the row and column scores are equidistant for successive categories is known as the *Uniform* (U) AM.

In case the RC model is not of adequate fit, multiplicative row-column AMs of higher order can be considered. Such a model of *M*-th order is defined as

(8) $$\log m_{ij}=\lambda +\lambda_{i}^{X}+\lambda_{j}^{Y}+\sum_{m=1}^{M}\zeta_{m}\mu_{im}\nu_{jm}\;,\;i=1,\ldots ,I\;,\;j=1,\ldots ,J\;,$$

with $1\leq M\leq M^{*}=\min\left(I,J\right)-1$, and denoted by RC(M). Model RC$\left(M^{*}\right)$ is an equivalent expression of the saturated model (2). The sum $\sum_{m=1}^{M}\zeta_{m}\mu_{im}\nu_{jm}$ in (8) corresponds to the generalized singular value decomposition of the matrix of interaction parameters of model (2), $\Lambda ={\left(\lambda_{ij}^{XY}\right)}_{I\times J}$, and *M* is the rank of Λ. The $\zeta_{m}$s are the associated eigenvalues, satisfying thus $\zeta_{1}\geq \ldots \geq \zeta_{M}>0$. Vectors $\mu_{m}\;=\;\left(\mu_{1m},\ldots ,\mu_{Im}\right)$ and $\nu_{m}\;=\;\left(\nu_{1m},\ldots ,\nu_{Jm}\right)$ are the corresponding row and column eigenvectors, m=1,…,M, which are orthonormalized with respect to the weights $w_{1}$ and $w_{2}$, i.e., the following constraints are satisfied:

(9) $$\begin{array}{ccc} \sum_{i}w_{1i}\mu_{im} & = & \sum_{j}w_{2j}\nu_{jm}=0,\;\;\;m=1,\ldots ,M\;,\\ \sum_{i}w_{1i}\mu_{im}\mu_{i\ell } & = & \sum_{j}w_{2j}\nu_{jm}\nu_{j\ell }=\delta_{m\ell },\;\;\;m,\ell =1,\ldots ,M, \end{array}$$

where $\delta_{m\ell }$ is Kronecker’s delta. It can easily be verified that df(RC(M))=(I−M−1)(J−M−1). AMs have been mainly developed by Goodman (see [4,5] and references therein) and are thus often referred to as Goodman’s AMs. For a detailed presentation of the association models, their inference, properties, interpretation, the role of the weights used and associated literature, we refer to the book of Kateri [7] (Chapter 6).

For ease in understanding but also for interpretation purposes, it is convenient to think in terms of the local associations of the table and define thus AMs through the local odds ratios (LORs)

(10) $$\theta_{ij}=\frac{\pi_{ij}\pi_{i+1,j+1}}{\pi_{i+1,j}\pi_{i,j+1}}\;,\;\;\;i=1,\ldots ,I-1,\;j=1,\ldots ,J-1.$$

Recall that the (I−1)×(J−1) table of LORs $\theta =(\theta_{ij})$, jointly with the marginal probabilities vectors $\pi_{r}$ and $\pi_{c}$, specify uniquely the corresponding I×J probability table π. Thus, given $\pi_{r}$ and $\pi_{c}$, a model on π can equivalently be expressed in terms of θ. Hence, model (8) is alternatively defined as

(11) $$\log\theta_{ij}=\sum_{m=1}^{M}\zeta_{m}\left(\mu_{im}-\mu_{i+1,m}\right)\left(\nu_{jm}-\nu_{j+1,m}\right)\;,$$

for i=1,…,I−1,j=1,…,J−1. In this set-up, the diffrences of successive row and column scores are constant for the U model, equal to

(12) $$\mu_{i}-\mu_{i+1}=\Delta_{1}\;\;\mathrm{and}\;\;\;\nu_{j}-\nu_{j+1}=\Delta_{2},\;\;i=1,\ldots ,I-1,\;j=1,\ldots ,J-1\;,$$

since scores for successive categories are equidistant. Hence, the U model is equivalently defined as

(13) $$\log\theta_{ij}=\zeta \left(\mu_{i}-\mu_{i+1}\right)\left(\nu_{j}-\nu_{j+1}\right)=\zeta \Delta_{1}\Delta_{2}=\log\theta =\theta^{\left(0\right)}\;$$

and is the model under which all local odds ratios are equal across the table, justifying its ‘uniform association’ characterization.

### 2.2. Correlation Models

A popular, mainly descriptive method for exploring the pattern of association in contingency tables is correspondence analysis (CA). The detailed discussion of CA is beyond our scope. For this, we refer to the book of Greenacre [8]. Correspondence analysis is a reparameterized version of the canonical correlation model of order *M*

(14) $$p_{ij}=p_{i+}p_{+j}\left(1+\sum_{m=1}^{M}\rho_{m}x_{im}y_{jm}\right),\;\;i=1\ldots ,I,\;j=1,\ldots ,J\;,$$

with $1\leq M\leq M^{*}$. The row and column scores ($x_{m}=\left(x_{1m},\ldots ,x_{Im}\right)$ and $y_{m}=\left(y_{1m},\ldots ,y_{Jm}\right)$, m=1,…,M) satisfy constraints (9) subject to the marginal weights. Usually, it is assumed M=2 and the row and column scores (coordinates) are graphically displayed as points in two-dimensional plots. The similarities between (8), expressed in terms of π in its multiplicative form, and (14) are obvious. Thus, motivated by the inferential approaches for AMs, MLEs have been considered for model (14), leading to the row-column correlation model of order *M*, while, for M=1, special correlation models of U, R or C type have also been discussed by Goodman [4,5].

## 3. ϕ-Divergence Based Association Models

AMs and correlation models were developed competitively and opposed to each other (cf. [5]), until Gilula et al. [9] linked them in an inspiring manner under an information theoretical approach. They proved that, under certain (common) conditions, both of them are the closest model to independence. Their difference lies on the divergence used for measuring their closeness to independence. AMs are the closest in terms of the KL divergence and correlation models in terms of the Pearson’s divergence. This result motivated subsequent research and led to the definition of general classes of dependence models by substituting the KL and Pearson divergences through generalized families of divergences.

Under the conditions of [9], namely for given marginal distributions ($\pi_{r}$ and $\pi_{c}$), given scores (μ and ν) and given their correlation ρ=corr(μ,ν), Kateri and Papaioannou [10] derived that the joint distribution π that is closest to independence in terms of the ϕ–divergence is of the form

(15) $$\pi_{ij}=\pi_{i+}\pi_{+j}F^{-1}\left(\alpha_{i}+\beta_{j}+\zeta \mu_{i}\nu_{j}\right),\;\;i=1,\ldots ,I,\;j=1,\ldots ,J\;,$$

where $F^{-1}$ is the inverse function of $F\left(x\right)=\phi^{\prime }\left(x\right)$. The scores μ and ν satisfy the constraints (7) with marginal weights. Under these constraints, it can easily be verified that $\rho =\mathrm{corr}\left(\mu ,\nu \right)=\sum_{i,j}\mu_{i}\nu_{j}\pi_{ij}$. Additionally, the identifiability constraints

(16) $$\sum_{i}\pi_{i+}\alpha_{i}=\sum_{j}\pi_{+j}\beta_{j}=0$$

are imposed on parameters $\alpha =(\alpha_{1},\ldots ,\alpha_{I}))$ and $\beta =(\beta_{1},\ldots ,\beta_{J}))$. The parameter ζ is measuring association. It can be verified that (15), due to (7) with marginal weights and (16), leads to

(17) $$\zeta \left(\pi ,\mu ,\nu \right)=\sum_{i,j}\pi_{i+}\pi_{+j}\mu_{i}\nu_{j}F\left(\frac{\pi_{ij}}{\pi_{i+}\pi_{+j}}\right)$$

and that ζ=0 if and only if the independence model holds ($\pi =\pi^{I}$). Furthermore, under model (15), the correlation ρ between the row and column scores is increasing in ζ and the ϕ-divergence measure $I_{\phi }^{C}\left(\pi ,\pi^{I}\right)$, for given ϕ-function, is increasing in |ζ|.

Model (15), with known row and column scores, is the ϕ–divergence based extension of the LL model and is denoted by LL${}_{\phi }$. If the scores are additionally equidistant for successive categories, (15) becomes the ϕ–divergence based U model, U${}_{\phi }$, while the classes of models R${}_{\phi }$, C${}_{\phi }$ and RC${}_{\phi }$ are defined analogously. The standard LL, R, C or RC models correspond to ϕ(x)=xlogx. The analogue correlation models, defined by (14) for M=1, are derived for $\phi \left(x\right)={\left(1-x\right)}^{2}$, setting $\mu =x_{1}$, $\nu =y_{1}$ and $\zeta =\rho_{1}$. For the standard association and correlation models, (17) simplifies to

(18) $$\zeta \left(\pi ,\mu ,\nu \right)=\sum_{i,j}\pi_{i+}\pi_{+j}\mu_{i}\nu_{j}\log\left(\pi_{ij}\right)$$

and

(19) $$\zeta \left(\pi ,\mu ,\nu \right)=\sum_{i,j}\mu_{i}\nu_{j}\pi_{ij}=\mathrm{corr}\left(\mu ,\nu \right)=\rho \;,$$

respectively.

For the power divergence (for $\phi \left(x\right)=\frac{x^{\lambda +1}-x}{\lambda (\lambda +1)}$, λ≠−1,0), model (15) becomes

(20) $$\pi_{ij}=\pi_{i+}\pi_{+j}{\left[\frac{1}{\lambda +1}+\lambda \left(\alpha_{1}+\beta_{j}+\zeta \mu_{i}\nu_{j}\right)\right]}^{1/\lambda },\;\;i=1,\ldots ,I,\;j=1,\ldots ,J\;,$$

considered by Rom and Sarkar [11]. Expression (20) defines parametric classes of AMs, controlled by the parameter λ, which are denoted by LL${}_{\lambda }$, U${}_{\lambda }$, R${}_{\lambda }$, C${}_{\lambda }$ or RC${}_{\lambda }$, according to the assumption made for the row and column scores.

The RC(M) model, $1\leq M\leq M^{*}$, is analogously generalized to RC${}_{\phi }\left(M\right)$, the class of ϕ–divergence AMs of order *M*, given by

(21) $$\pi_{ij}=\pi_{i+}\pi_{+j}F^{-1}\left(\alpha_{i}+\beta_{j}+\sum_{m=1}^{M}\zeta_{m}\mu_{im}\nu_{jm}\right),\;\;i=1,\ldots ,I,\;j=1,\ldots ,J\;,$$

where the scores $\mu_{m}$ and $\nu_{m}$ satisfy restrictions (9) with marginal weights. The standard RC(*M*) model (8) is derived for ϕ(x)=xlogx, while, for $\phi \left(x\right)={\left(1-x\right)}^{2}$, model (21) becomes the correlation model (14) with $\mu_{m}=x_{m}$, $\nu_{m}=y_{m}$ and $\zeta_{m}=\rho_{m}$, m=1,…,M.

Models (15) and (21) can alternatively be expressed as

(22) $$\theta_{ij}^{\left(\phi \right)}=\zeta \left(\mu_{i}-\mu_{i+1}\right)\left(\nu_{j}-\nu_{j+1}\right),\;\;i=1,\ldots ,I-1,\;j=1,\ldots ,J-1\;,$$

and

(23) $$\theta_{ij}^{\left(\phi \right)}=\sum_{m=1}^{M}\zeta_{m}\left(\mu_{im}-\mu_{i+1,m}\right)\left(\nu_{jm}-\nu_{j+1,m}\right),\;\;i=1,\ldots ,I-1,\;j=1,\ldots ,J-1\;,$$

respectively, where $\theta_{ij}^{\left(\phi \right)}$ is a scaled measure of local dependence, defined for i=1,…,I−1, j=1,…,J−1 as

(24) $$\theta_{ij}^{\left(\phi \right)}\left(\pi \right)=F\left(\frac{\pi_{ij}}{\pi_{i+}\pi_{+j}}\right)+F\left(\frac{\pi_{i+1,j+1}}{\pi_{i+1,+}\pi_{+,j+1}}\right)-F\left(\frac{\pi_{i+1,j}}{\pi_{i+1,+}\pi_{+j}}\right)-F\left(\frac{\pi_{i,j+1}}{\pi_{i+}\pi_{+,j+1}}\right).$$

For ϕ(x)=xlogx, (24) becomes the well-known log local odds ratio $\log(\theta_{ij})$, modelled in (13) and from now on denoted as $\theta_{ij}^{\left(0\right)}=\log\left(\theta_{ij}\right)$. For the power divergence, we get

(25) $$\theta_{ij}^{\left(\lambda \right)}\left(\pi \right)=\frac{1}{\lambda }\left[{\left(\frac{\pi_{ij}}{\pi_{i+}\pi_{+j}}\right)}^{\lambda }+{\left(\frac{\pi_{i+1,j+1}}{\pi_{i+1,+}\pi_{+,j+1}}\right)}^{\lambda }-{\left(\frac{\pi_{i+1,j}}{\pi_{i+1,+}\pi_{+j}}\right)}^{\lambda }-{\left(\frac{\pi_{i,j+1}}{\pi_{i+}\pi_{+,j+1}}\right)}^{\lambda }\right].$$

**Remark** **1.** *Kateri and Papaioannou [10] studied properties of the class of ϕ-divergence based AMs. Additionally, they developed a test based on a ϕ-divergence statistic for testing the GOF of ϕ-divergence AMs and studied its efficiency. The choice of the ϕ-function in the test statistic is independent of the ϕ-function used for the model definition. Thus, it serves as a ϕ-divergence based GOF test for the traditional well-known association or correlation models.*

In order to understand the role and nature of the scaled measures of local association (24), one can examine a simple 2×2 contingency table. In this case, (10) becomes the well-known odds ratio $\theta \;(=\;\theta_{11})$. Its ϕ-divergence scaled generalization, (24) for I=J=2, has been explored by Espendiller and Kateri [12]. For these ϕ-scaled association measures for 2×2 tables, asymptotic tests of significance and confidence intervals (CIs) are constructed based on the fact that they are asymptotically normal distributed. An interesting feature of the family of ϕ-scaled association measures for 2×2 tables is that when the odds ratio θ cannot be finitely estimated (due to sampling zeros), some members of this family provide finite estimates, while, for a subset of them, their variance is also finite. Extensive evaluation studies verified earlier statements in the literature about the low coverage of the log odds ratio CIs when the association is very high (logθ>4). In such cases, focusing on the power divergence scaled odds ratios $\theta^{\left(\lambda \right)}$, $\theta^{\left(1/3\right)}$ for λ=1/3 is to be preferred, since the corresponding CI is of better coverage when approaching the borders of the parameter space and is in general less conservative than the classical log odds ratio CI [12]. Here, the role of the scale effect on measuring the local dependence will be further clarified in the examples discussed in Section 5.

**Remark** **2.** *The idea of viewing a model as a departure from a parsimonious reference model, with the property of being the closest to this reference model under certain conditions in terms of the KL divergence, can be adopted for other types of models as well, such as the quasi symmetry (QS) model and the logistic regression, lightening thus a different interpretational aspect of these models. Substitution of the KL divergence by the ϕ-divergence, leads further, in an analogous manner to AMs, to the derivation of generalized ϕ-divergence based classes of QS models [13], ordinal QS [14] and logistic regression models [15]. For example, in case of the QS, the role of the scaled measures (24) take the analogously defined scaled deviations from the model of complete symmetry.*

## 4. Uniform Local Association

We shall focus on the case of uniform local association and the corresponding ϕ divergence scaled model U${}_{\phi }$, defined by (15) or (22) with row and column scores satisfying (12). Model U${}_{\phi }$ is equivalently expressed as

(26) $$\theta_{ij}^{\left(\phi \right)}=\zeta \Delta_{1}\Delta_{2}=\theta^{\left(\phi \right)}\;,\;\;i=1,\ldots ,I-1,\;j=1,\ldots ,J-1\;$$

and forms a family of models. Compare to (13) for the U model defined in terms of the usual log odds ratio. The associated probability table under model U${}_{\phi }$ is uniquely specified by the one-to-one map $\left(\pi_{r},\pi_{c},\theta^{\left(\phi \right)}\right)\mapsto \pi^{U_{\phi }}$, not given in a closed-form expression.

The MLEs of the marginal probabilities are the corresponding marginal sample proportions ${\hat{\pi }}_{r}=p_{r}={\left(p_{1+},\ldots ,p_{I+}\right)}^{T}$, ${\hat{\pi }}_{c}=p_{c}={\left(p_{+1},\ldots ,p_{+J}\right)}^{T}$, while the MLE of $\theta^{\left(\phi \right)}$, ${\hat{\theta }}^{\left(\phi \right)}$, is not available in explicit form. For a given ϕ-function, model U${}_{\phi }$ belongs to the family of homogeneous linear predictor (HLP) models [16]. It is straightforward to verify that it satisfies the two conditions of Definition 3 in [16]. In practice, it can be fitted using Lang’s mph R-package. The standard U model, denoted in the sequel as U${}_{0}$, has the equivalent HLP model expression

(27) $$L\left(m_{v}\right)=C\log\left(m_{v}\right)=X\beta =1_{\left(I-1)(J-1\right)}\theta^{\left(0\right)}\;,$$

where X and β are the model’s design matrix and parameter vector (scalar for U${}_{0}$), respectively. Furthermore, $\theta^{\left(0\right)}=\log\left(\theta \right)$, $m_{v}$ is the IJ×1 vector of expected cell frequencies, corresponding to the I×J table m expanded by rows, and C is an (I−1)(J−1)×IJ design matrix so that the vector of all log(LOR)s is derived, i.e., $C\log\left(m_{v}\right)={\left(\theta_{11}^{\left(0\right)},\ldots ,\theta_{I-1,J-1}^{\left(0\right)}\right)}^{T}$. For more details on inference for model U${}_{0}$ through HLP models, we refer to [7] (Sections 5.6 and 6.6.4). In Section 6.6.4 of [7], the approach is implemented in R for the example of Table 1 (see Section 5), while an R-function for constructing the design matrix C for two-way tables of any size is provided in the web appendix of the book. This approach is easily adjusted for model U${}_{\phi }$, by replacing $\log(m_{v})$ in (27) by $F(m_{vs})$, where $m_{vs}$ is the IJ×1 vector with entries $\frac{m_{ij}}{m_{i+}m_{+j}/n}=\frac{\pi_{ij}}{\pi_{i+}\pi_{+j}}$, expanded by rows.

Under U${}_{\phi }$, all 2×2 subtables, formed by any successive rows and any successive columns, share the same ϕ-scaled local association $\theta^{\left(\phi \right)}$. It is of practical interest to have estimators of this common local association, alternative to the MLEs, that are provided in explicit forms. One such estimator, based on the sample version of (17), is given by

(28) $${\tilde{\theta }}^{\left(\phi \right)}=\zeta \left(p,\mu ,\nu \right)\Delta_{1}\Delta_{2}=\Delta_{1}\Delta_{2}\sum_{i,j}p_{i+}p_{+j}\mu_{i}\nu_{j}F\left(\frac{p_{ij}}{p_{i+}p_{+j}}\right).$$

Another option is

(29) $${\bar{\theta }}^{\left(\phi \right)}=\frac{1}{\left(I-1)(J-1\right)}\sum_{i=1}^{I-1}\sum_{j=1}^{J-1}\theta_{ij}^{\left(\phi \right)}\left(p\right)\;,$$

which is the mean of the sample ϕ-scaled local association measures (24). For the power-divergence based models U${}_{\lambda }$, estimators (28) and (29) are denoted by ${\tilde{\theta }}^{\left(\lambda \right)}$ and ${\tilde{\theta }}^{\left(\lambda \right)}$, respectively. For the U correlation model, derived for λ=1 and denoted thus by U${}_{1}$, estimator (28) takes the form

(30) $${\tilde{\theta }}^{\left(1\right)}=\Delta_{1}\Delta_{2}\sum_{i,j}\mu_{i}\nu_{j}p_{ij}=\Delta_{1}\Delta_{2}r,$$

where *r* is the sample correlation between the row and column scores (compare to (19)).

Under the U${}_{\phi }$ model, $\theta^{\left(\phi \right)}$ is the single association parameter, measuring the strength of the local association that is uniform across the table. Furthermore, $\theta^{\left(\phi \right)}=0\left(\Leftrightarrow \zeta =0\right)$ if and only if the independence (I) model holds. Since model I is nested in U${}_{\phi }$, the following test of independence, conditional on U${}_{\phi }$, can be considered

(31) $$G^{2}\left(I|U_{\phi }\right)=G^{2}\left(I\right)-G^{2}\left(U_{\phi }\right)\;,$$

which is asymptotically $X_{1}^{2}$ distributed. This test is well-known for the standard U${}_{0}$ model (see [17] and Section 6.3 in [7]).

**Remark** **3.** *For model U${}_{0}$, Tomizawa [18] proposed a measure of divergence from uniform association, based on the KL-divergence, taking values in the interval [0,1) and being equal to 0 if and only if U${}_{0}$ holds. He constructed also asymptotic confidence interval for this measure, provided U${}_{0}$ does not hold. Conde and Salicrú [19] extended his work by considering such a measure based on the ϕ-divergence and developed asymptotic inference for it, and also for the case that U${}_{0}$ holds. Their approach and measures should not be confused with the approach followed here. They aim at detecting departures from U${}_{0}$ (in favor of a non-uniform association structure) while here we focus on measuring the strength of uniform association, provided that U${}_{0}$ (or U${}_{\phi }$) holds.*

Tomizawa [18] as well as Conde and Salicrú [19] based the estimation of their measures on the following closed-form estimator of $\theta^{\left(0\right)}$ under U${}_{0}$

(32) $$\theta_{*}^{\left(0\right)}=\log\left(\frac{\sum_{i=1}^{I-1}\sum_{j=1}^{J-1}\theta_{ij}^{\left(0\right)}\left(p\right)}{\left(I-1)(J-1\right)}\right)\;.$$

**Remark** **4.** *For square contingency tables with commensurable classification variables, analogous to the measure of departure from U${}_{0}$ (see Remark 3), Tomizawa et al. [20] introduced a measure of departure from complete symmetry relying on the power divergence and Tomizawa [21] a measure of departure from marginal homogeneity. Kateri and Papaioannou [22] extended these measures to corresponding ϕ-divergence based measures and proposed further ϕ-divergence based measures of departure from the QS and triangular symmetry models. The work of Menéndez et al. [23,24] is also related.*

## 5. Illustrations

We revisit a classical data set of Grizzle [25], provided in Table 2, which is adequately modelled by the U${}_{0}$ model (see [18,19]). Our second data set in Table 1 corresponds to a study in [26] and provides strong evidence in favor of U${}_{0}$ (see [7]). The maximum likelihood estimates of the expected cell frequencies under U${}_{0}$ are also given in parentheses in Table 1 and Table 2.

The $G^{2}$ test statistics for the U${}_{\lambda }$ models fitted on these data, for λ→0 and λ=1/3, 2/3, 1 are provided in Table 3, along with corresponding ${\hat{\theta }}^{\left(\lambda \right)}$, ${\tilde{\theta }}^{\left(\lambda \right)}$ and ${\bar{\theta }}^{\left(\lambda \right)}$ values, i.e., the estimates of the $\theta^{\left(\lambda \right)}$s discussed in Section 4. For U${}_{0}$, the $\theta_{*}^{\left(0\right)}$ estimates (32) for Table 1 and Table 2 are equal to 0.2890 and 0.7972, respectively.

We observe that all considered U${}_{\lambda }$ models are of very similar (acceptable) fit for the first example while they differ enormously for the second one. For the data of Table 1, the fit of U${}_{0}$ is impressive, that of U${}_{1/3}$ acceptable while U${}_{2/3}$ and U${}_{1}$ are of very bad fit. A reverse situation appears for another data set, given in Table 4. In this case model, U${}_{1}$ can be accepted while U${}_{0}$ can not (see Table 5). The maximum likelihood estimates of the expected cell frequencies under U${}_{1}$ are provided in Table 4 in parentheses.

Observe (in Table 3) that the closed form estimates for the λ-scaled local associations are close to the corresponding maximum likelihood estimates in case the assumed model is of adequate fit while they diverge for models of bad fit.

## 6. Conclusions

We revealed the role of ϕ-divergence in modelling association in two-way contingency tables and illustrated it for the special case of uniform association in ordinal contingency tables. Targeting at pointing out the potential of this modelling approach and the generated families of models, we avoided presenting technical details, properties and inferential results for these models, which can be found in the initial sources cited.

Crucial quantities are the $\theta_{ij}^{\left(\phi \right)}$s, the ϕ-scaled measures of local association. The generalized family of ϕ-divergence based AMs enriches the modelling options in CTA, since the pattern of underlying association structure in a table may be simplified and thus described by a more parsimonious model when considering a different scale. A crucial issue, as also pointed out by one of the reviewers, is how to decide on the scale. So far, such a decision is based on trials of various alternative options. A formal approach selecting the scale is missing. In case of a parametric family, like the one based on the power divergence, the problem can be tackled by considering λ as an unknown parameter and estimating it from the data. Such an approach has been followed in the logistic regression set-up by Kateri and Agresti [15].

It is important to realize that, due to the scale difference, $\theta_{ij}^{\left(\phi \right)}$s are not directly comparable for different ϕ-function (or λ-values in case of the power divergence). Thus, comparisons across different ϕs (or λs) are possible only in terms of the corresponding expected cell frequencies or a common measure of local association evaluated on them. AMs can also be considered for other types of generalized odds ratios, like, for example, the global odds ratios. The extension of such models through the ϕ-divergence and their study is the subject of a work in progress. Inference for closed form estimators of the common $\theta^{\left(\phi \right)}$ of the U${}_{\phi }$ model and comparisons among them is the content of a paper under preparation.

The conditional test of independence (31) can be based not only on U${}_{\phi }$ but on LL${}_{\phi }$ models as well. Another 1 df test of independence for ordinal classification variables is the linear trend test of Mantel (see [27]). It considers the testing problem $H_{0}:\;\rho =0$ vs. $H_{1}:\;\rho \neq 0$, where ρ is the correlation between ordered scores assigned to the categories of the classification variables of the table. It is thus applicable only when the underlying association exhibits a linear trend for the assigned scores. The test of Mantel uses the test statistic $M^{2}=\left(n-1\right)r^{2}$, which is under $H_{0}$ asymptotically $X_{1}^{2}$ distributed. The way this test is linked to the above-mentioned conditional tests, in view also of (19), is interesting to be investigated further.

Throughout this paper, we assumed a multinomial sampling scheme. For the models considered here, the other two classical sampling schemes for contingency tables (independent Poisson and product multinomial) are inferentially equivalent. Furthermore, for ease of presentation, we restricted here to two-way tables. The proposed models extend straightforwardly to multi-way tables. For two- or higher-dimensional tables, the subset of models that are linear in their parameters (i.e., RC- and RC(M)-type terms are excluded) belong to the family of homogeneous linear predictor (HLP) models [16] and can thus be fitted using the R-package mph.

## Figures and Tables

**Table 1:** Students’ survey about cannabis use at the University of Ioannina, Greece (1995). The maximum likelihood estimates of the expected cell frequencies under the U${}_{0}$ model are given in parentheses.

Alcohol Consumption	I Tried Cannabis...			
	Never	Once or Twice	More Often	Total
at most once/month	204 (204.4)	6 (5.7)	1 (0.9)	211
twice/month	211 (211.4)	13 (13.1)	5 (4.5)	229
twice/week	357 (352.8)	44 (48.8)	38 (37.4)	439
more often	92 (95.3)	34 (29.4)	49 (50.3)	175
Total	864	97	93	1054

**Table 2:** Cross-classification of duodenal ulcer patients according to operation and dumping severity. The maximum likelihood estimates of the expected cell frequencies under the U${}_{0}$ model are given in parentheses.

Operation	Dumping Severity			
	None	Slight	Moderate	Total
A	61 (62.5)	28 (26.2)	7 (7.3)	96
B	68 (62.9)	23 (30.9)	13 (10.2)	104
C	58 (61.0)	40 (35.3)	12 (13.7)	110
D	53 (53.7)	38 (36.6)	16 (16.7)	107
Total	240	129	48	417

**Table 3:** Goodness of fit of the U${}_{\lambda }$ models for the data of Table 1 and Table 2 along with estimates of the common λ-scaled local association $\theta^{\left(\lambda \right)}$ under U${}_{\lambda }$.

**Example in Table 1**		
λ	$G^{2}$ (*p*-value)	${\hat{\theta }}^{\left(\lambda \right)}$/${\tilde{\theta }}^{\left(\lambda \right)}$/${\bar{\theta }}^{\left(\lambda \right)}$
0	1.47 (0.917)	0.8026/0.7817/0.7814
1/3	7.19 (0.207)	0.6857/0.6451/0.6432
2/3	25.21 (0.000)	0.4720/0.5853/0.5974
1	40.76 (0.000)	0.3667/0.5732/0.6096
**Example in Table 2**		
λ	$G^{2}$ (*p*-value)	${\hat{\theta }}^{\left(\lambda \right)}$/${\tilde{\theta }}^{\left(\lambda \right)}$/${\bar{\theta }}^{\left(\lambda \right)}$
0	4.59 (0.468)	0.1626/0.1665/0.1612
1/3	4.57 (0.471)	0.1619/0.1638/0.1573
2/3	4.55 (0.473)	0.1612/0.1616/0.1541
1	4.52 (0.477)	0.1606/0.1599/0.1515

**Table 4:** Hypothetical 4×3 data table with maximum likelihood estimates of the expected cell frequencies under U${}_{1}$ (in parentheses).

160 (159.5)	8 (7.8)	1 (1.4)
198 (199.4)	16 (14.7)	14 (13.9)
310 (321.8)	40 (32.9)	50 (45.4)
161 (149.1)	12 (20.4)	30 (33.8)

**Table 5:** Goodness of fit of the U${}_{\lambda }$ models and maximum likelihood estimates of the common λ-scaled local association $\theta^{\left(\lambda \right)}$ under U${}_{\lambda }$ for the data of Table 4.

λ	$G^{2}$ (*p*-Value)	${\hat{\theta }}^{\left(\lambda \right)}$
0	15.99 (0.007)	0.3082
1/3	13.52 (0.019)	0.3266
2/3	10.64 (0.059)	0.3391
1	7.95 (0.159)	0.3215

## Abbreviations

The following abbreviations are used in this manuscript:

CTA	contingency table analysis
MLE	maximum likelihood estimator
MϕEs	minimum ϕ-divergence estimators
KL	Kullback–Leibler
GOF	goodness of fit
I	independence model
AMs	association models
LL model	linear by linear association model
R model	row effect association model
C model	column effect association model
RC model	multiplicative row-column effect association model
U model	uniform association model
df	degrees of freedom
LOR	local odds ratio
CA	correspondence analysis
CI	confidence interval
QS	quasi symmetry
HLP	homogeneous linear predictor
